# Molecular Imaging of Steroid-Induced Osteonecrosis of the Femoral Head through iRGD-Targeted Microbubbles

**DOI:** 10.3390/pharmaceutics14091898

**Published:** 2022-09-08

**Authors:** Ping Zhao, Shuai Zhao, Jiaqi Zhang, Manlin Lai, Litao Sun, Fei Yan

**Affiliations:** 1 Department of Ultrasound, The First Affiliated Hospital, Guangzhou University of Chinese Medicine, Guangzhou 510407, China; 2 Department of Ultrasound, Suzhou Hospital of Anhui Medical University (Suzhou Municipal Hospital of Anhui Province), Suzhou 234000, China; 3 Department of Ultrasound, The Second People’s Hospital of Shenzhen, The First Affiliated Hospital of Shenzhen University, Shenzhen 518061, China; 4 Cancer Center, Department of Ultrasound Medicine, Zhejiang Provincial People’s Hospital (Affiliated People’s Hospital), Hangzhou Medical College, Hangzhou 310014, China; 5 Center for Cell and Gene Circuit Design, CAS Key Laboratory of Quantitative Engineering Biology, Shenzhen Institute of Synthetic Biology, Shenzhen Institutes of Advanced Technology, Chinese Academy of Sciences, Shenzhen 518055, China

**Keywords:** osteonecrosis of the femoral head, ultrasound molecular imaging, microbubbles, integrin α_v_β_3_, iRGD peptides

## Abstract

Osteonecrosis of the femoral head (ONFH) is a disease that is commonly seen in the clinic, but its detection rate remains limited, especially at the early stage. We developed an ultrasound molecular imaging (UMI) approach for early diagnosis of ONFH by detecting the expression of integrin α_v_β_3_ during the pathological changes in steroid-induced osteonecrosis of the femoral head (SIONFH) in rat models. The integrin α_v_β_3_-targeted PLGA or lipid microbubbles modified with iRGD peptides were fabricated and characterized. Their adhesion efficiency to mouse brain microvascular endothelial cells in vitro was examined, and the better LIPO_iRGD_ was used for further in vivo molecular imaging of SIONFH rats at 1, 3 and 5 weeks; revealing significantly higher UMI signals could be observed in the 3-week and 5-week SIONFH rats but not in the 1-week SIONFH rats in comparison with the non-targeted microbubbles (32.75 ± 0.95 vs. 0.17 ± 0.09 for 5 weeks, *p* < 0.05; 5.60 ± 1.31 dB vs. 0.94 ± 0.81 dB for 3 weeks, *p* < 0.01; 1.13 ± 0.13 dB vs. 0.73 ± 0.31 dB for 1 week, *p* > 0.05). These results were consistent with magnetic resonance imaging data and confirmed by immunofluorescence staining experiments. In conclusion, our study provides an alternative UMI approach to the early evaluation of ONFH.

## 1. Introduction

Osteonecrosis of the femoral head (ONFH) is a disease commonly seen in the clinic and has a low detection rate and high disability rate, especially in the early stage [1]. Generally, ONFH is divided into two types: traumatic osteonecrosis of the femoral head and non-traumatic osteonecrosis of the femoral head [2]. Among the non-traumatic ONFH, steroid-induced osteonecrosis of the femoral head (SIONFH) has received extensive attention due to the difficulty of restoring osteonecrotic tissue [3,4]. Although the exact pathologic process of this disease is still less understood, the development of the disease may cause irreversible damage to the femoral head, which seriously influences the patient’s quality of life [5]. Usually, SIONFH is caused by long-term or high-dose use of glucocorticoids, leading to disturbance of microcirculation perfusion in the femoral head and the sequential death of bone cells and bone marrow cells [6,7]. Early detection and intervention in patients with SIONFH are important for these patients to retain their quality of life.

In the early stage, SIONFH is characterized by osteolytic bone destruction with enhanced bone resorption and granulation tissue with a rich blood supply that performs the repair reaction to bring about normalization of blood vessels [8,9,10]. This stage is the key process in the occurrence and development of ONFH. Accurate evaluation of these pathological features plays an important role in the diagnosis and prognosis of the disease [10,11]. Magnetic resonance angiography (MRA) is the most widely used approach, having some advantages such as free contrast agent and non-radiation [12]. However, MRA makes it difficult to detect the blood supply in the femoral head, and distortion artifacts in low-speed blood vessels and those with eddy currents may occur [13]. Part of the MRA signal is usually lost, and small-scale vascular structures and lesions cannot be accurately displayed [14,15]. CT angiography (CTA) and digital subtraction angiography (DSA) require an iodine contrast medium, and this technique involves exposure to radiation [16,17]. Additionally, it is difficult to justify them as the first choice for screening patients in the asymptomatic stage [18,19]. By contrast, contrast-enhanced ultrasound (CEUS) is more convenient for vascular examination. With this technique, the ultrasound contrast agent (UCA), excited by appropriate acoustic energy, oscillates to produce a large number of backscatter signals, enhancing the reflected ultrasound signal and decreasing the surrounding tissue background signal [20,21]. More importantly, by designing and using the targeted contrast agents, the expression level and spatial distribution of some key biomarkers on the surface of the vascular endothelium in the ONFH could be observed by ultrasound imaging, providing more accurate judgment for diagnosis, treatment and prognosis [22,23].

The literature has demonstrated that SIONFH contains a large amount of new granulation tissue and rich blood vessels in the process of ischemia-necrosis repair [24,25,26,27]. Among these, integrin α_v_β_3_ on the surface of neovascular endothelial cells takes an important role in the occurrence and development of SIONFH. Evidence demonstrates that integrins can mediate the repair of articular cartilage through a series of mechanisms, including inhibiting the degradation of the extracellular matrix, increasing the synthesis of the extracellular matrix, inhibiting the apoptosis of chondrocytes, and promoting the proliferation and migration of chondrocytes [28]. Integrin α_v_β_3_, highly expressed on the surface of osteoblasts, can also mediate the migration and adhesion of osteoclasts to the exposed bone surface or promote osteoblast adhesion, proliferation and mineralization [29,30]. Considering the high expression of integrin receptor α_v_β_3_ on the surface of neovascular endothelial cells and its important role in the occurrence and development of SIONFH [31], in this study, we developed integrin-targeted microbubbles and used them for detecting the expression of integrin α_v_β_3_ on the surface of neovascular endothelial cells by ultrasound molecular imaging (UMI) to dynamically and non-invasively monitor the pathological process of necrosis and repair of SIONFH.

## 2. Materials and Methods

### 2.1. Preparation of Targeted LIPO_iRGD_ and PLGA_iRGD_

The synthesis of DSPE-PEG2000-iRGD was based on previous reports [32]. In brief, DSPE-PEG2000-iRGD was prepared by the dialysis-freeze-drying method using DSPE-PEG2000-maleimide (Jinsui Bio-Technology, Shanghai, China) and CCRGDKGPDC-NH_2_ (GL Biochem, Shanghai, China) (molar ratio 1.0/1.5) according to a previous report. All chemical reagents had a purity above 95%.

The α_v_β_3_-integrin targeted lipid microbubbles (LIPO_iRGD_) were prepared by mixing 1,2-distearoyl-sn-glycero-3-phosphocholine (DSPC; Avanti Polar Lipids, Alabaster, AL, USA) with DSPE-PEG2000/DSPE-PEG2000-iRGD (molar ratio = 9/0.5) in chloroform, followed by volatilization with a low flow rate of nitrogen for more than 3 h in a vacuum at room temperature. The dried mixture was hydrated with Tris buffer solution (0.1 M, PH = 7.4) at 65 °C. Then, the air in the bottle was replaced with C_3_F_8_ (FluoroMed, Newport, TN, USA). LIPO_iRGD_ was finally obtained by mechanical vibration for 45 s. The α_v_β_3_ integrin-targeted PLGA microbubbles (PLGA_iRGD_) were prepared using the water/oil/water (W/O/W) double emulsion method [33]. Briefly, poly(lactic-co-glycolic acid) (PLGA; 50 mg), DSPC (2.5 mg) and DSPE-PEG2000-iRGD (0.5 mg) were dissolved in 1 mL of dichloromethane. After complete dissolution, 200 µL of NH_4_HCO_3_ (60 mg/mL) was added, and the solution was initially emulsified in an ice bath using an ultrasonic crusher (power: 130 kw; duration: 2 min; amplitude 40–50%; pulse: 3 s on, 3 s off); then, 5 mL of 4% polyvinyl alcohol (PVA) solution was added to form the W/O/W solution. The solution was stirred in a magnetic agitator for 4–8 h to volatilize the dichloromethane, followed by centrifugation (1600× *g*, 10 min) and three rinses with deionized water. C_3_F_8_ gas was added to the freeze-dryer for 1 min at a rate of 50 mL/min to form PLGA_iRGD_. The freeze-dried PLGA_iRGD_ powder was stored in a refrigerator at 4 °C until use. Non-targeted LIPO_iRGD_ and PLGA_iRGD_ were also prepared according to the above-mentioned methods without adding DSPE-PEG2000-iRGD.

### 2.2. Characterization of LIPO_iRGD_ and PLGA_iRGD_

Ten microliters of the DiO or DiI working solution (100 ng/mL) was added to microbubble suspensions and rotated at a low speed (30 g) on ice for 30 min. After diluting with PBS buffer, the targeted LIPO_iRGD_ was subjected to low-speed centrifugal flotation (400× *g*, 4 min) three times to remove the free DiO or DiI in the supernatant, and the DiI-labeled targeted LIPO_iRGD_ and DiO-labeled PLGA_iRGD_ were obtained. The particle size, particle size distribution and concentrations of LIPO_iRGD_ and PLGA_iRGD_ were measured using an Accusizer 780 optical particle size analyzer (Particle Sizing Systems, Santa Barbara, CA, USA). Twenty microliters of the two types of MB suspension were also placed on glass slides and observed under a Confocal Fluorescence Laser Microscope (Nikon Instruments, Tokyo, Japan).

### 2.3. Cell Culture

bEnd.3 cells were cultured overnight in DMEM high glucose medium containing 10% fetal bovine serum and 1% penicillin-streptomycin solution on confocal culture dishes (1 × 10^5^ cells per well) at 37 °C in a 5% CO_2_ incubator. Experiments of the static adhesion of LIPO_iRGD_ and PLGA_iRGD_ to cells were carried out. Briefly, after the cells were cultivated overnight, the culture medium was removed and the targeted microbubbles (1 × 10^8^ bubbles/mL) were added to the cell monolayer for 5 min at room temperature. Free microbubbles were removed by rinsing with PBS. The white light images of microbubbles binding to cells were observed with a confocal microscope. To determine the binding specificity of the targeted microbubbles, the monolayer was blocked with excess iRGD free polypeptide (60 μg/mL) before the targeted microbubbles were added. The number of attached microbubbles was measured by confocal fluorescence microscopy under five random visual fields. 

### 2.4. Establishment of ONFH Model

All procedures using experimental animals have been approved by the management group of experimental animal nursing institutions of the technical research institute of Shenzhen Advanced Institute of Chinese Academy of Sciences. Male Sprague Dawley rats (weighing 100–150 g, age of 5–6 weeks) were purchased from Guangdong medical experimental animal center. All rats were fed alternately in a specific pathogen-free animal room with a 12:12 h light–dark cycle. The ONFH model was established by intravenous injection of lipopolysaccharide (40 μg/kg) at 24 h intervals. After 72 h, methylprednisolone (40 mg/kg) was injected into the gluteal muscle every 24 h, and penicillin (40U) was injected into the contralateral gluteal muscle to prevent infection [34,35]. Magnetic resonance imaging (MRI) was used to assess the successful establishment of ONFH after 1, 3 and 5 weeks. All the parameters are kept unchanged as follows: T1WI (TR = 608.0 ms, TE = 14.9 ms), T2WI (TR = 5156.0 ms, TE = 104.0 ms) and T2WI-FS (TR = 5156.0 ms, TE = 104.0 ms). After that, the rats with ONFH were selected for ultrasound molecular imaging.

### 2.5. In Vivo Ultrasound Molecular Imaging

Throughout the ultrasound imaging process, rats were anesthetized with 2% isoflurane in oxygen (2 L/min) on a heating pad. After shaving the thigh fur of rats, the ultrasonic gel was used as a coupling agent on the exposed skin of rats. Ultrasound molecular imaging was performed by a Mindray Resona 7 equipped with an L11-3U linear array probe. The parameters were kept unchanged in all imaging processes (gain: 65 dB, focal length: 2 mm, transmit power: 1.82%, mechanical index: 0.085). The region of interest (ROI) of the femoral head was manually outlined using the time–intensity curve (TIC) analysis software built into Mindray Resona 7. A fixed bracket was used to keep the probe in place throughout the inspection process. The location of the femoral head of the rat was identified in the two-dimensional B-mode images, and the whole femoral head was visible in the middle of the imaging field. The targeted or non-targeted MBs were injected through the tail vein of the rats. The method of breakdown/reperfusion was used to distinguish the targeted MB signals adhering to integrin α_v_β_3_ from the free non-targeted MB signals in blood circulation and tissue background signals. Briefly, after injecting the MBs, the ultrasound apparatus commenced recording images. After 4 min, about 100 frames of rat femoral head were recorded. Then the targeted or non-targeted MBs in the visual field were destroyed using a high-power ultrasonic sequence (transmission power: 100%, mechanical index: 0.787). After that, another 100 frame images were collected. To minimize signal bias from the adhering targeted MBs, we injected iRGD-targeted MBs and non-targeted MBs into the same rat in a random sequence, allowing for a waiting time of 30 min to remove MBs from the previous injection. The post-destruction acoustic signals were subtracted from the pre-destruction signals. The difference in acoustic signal intensity between pre-destruction and post-destruction ultrasonographic frames was calculated.

### 2.6. Image Data Analysis

The enhanced ROI area in the femoral head was drawn manually by the TIC analysis software built into Mindray Resona 7. The signal before destruction consisted of three parts: (a) the targeted MB signals adhering to integrin α_v_β_3_ receptor, (b) the free non-targeted MB signals in blood and (c) the background signal from imaging tissue in the visual field. After breakdown, the perfusion balance signal consisted of two parts: (d) the free non-targeted MB signal in blood and (e) the background signal from imaging tissue in the visual field. The average value of the signals before and after the destruction was calculated from the TIC, and the difference between them represented the targeted MB signals adhering to the integrin α_v_β_3_ receptor, which was displayed as a color overlay on the B-mode image.

### 2.7. Immunofluorescence Staining

The rats were euthanized immediately after the ultrasound molecular imaging experiment, and the bilateral femurs were removed for histological study. Double immunofluorescence staining of CD31 and integrin α_v_β_3_ was used to confirm the co-localization of integrin α_v_β_3_ on neovascular endothelial cells during osteonecrosis of the femoral head in rats. In brief, the tissue was cut into 6 μm sections by a freezing microtome. The slices were first incubated in a H_2_O_2_ (30%) solution to avoid light for 20 min and then placed in a citric acid antigen repair buffer (PH = 6.0) for antigen repair in a microwave oven for 20 min. After that, the sections were added to the autofluorescence quenching agent for 5 min and then washed with running water for 10 min. For serum blocking for 30 min, 3% BSA was added. The primary antibody (1:200 dilution) was dropped onto the sections and incubated overnight at 4 °C. The florescence-labeled secondary antibody (1:500 dilution) was added to the sections and incubated at room temperature in the dark for 50 min. After the sections were slightly dried, DAPI dye was added and incubated in the dark for 10 min at room temperature. After the slides were washed with PBS 3 times, 5 min each time, they were sealed with anti-fluorescence quenching agent (ethylenediamine tetraacetic acid) and examined under a fluorescence microscope.

### 2.8. Statistical Analysis

SPSS software (version 26, IBM Corp., Armonk, NY, USA) was used for statistical analysis. The quantitative data were expressed as mean ± standard deviation and were normally distributed with homogeneity of variance. The independent sample *t*-test was used for the comparison between groups. If the variance was inhomogeneous, the *t*-test was used. For data that satisfied a normal distribution and the variance was homogeneous, a variance analysis was used to compare the differences in multiple samples. The comparison between groups was tested by the Bonferroni method (bilateral test). *p* < 0.05 indicated a statistically significant difference between groups, while *p* < 0.01 was considered to indicate a very significant difference.

## 3. Results

### 3.1. Preparation and Characterization of iRGD-Targeted and Control MBs

**Figure 1:**
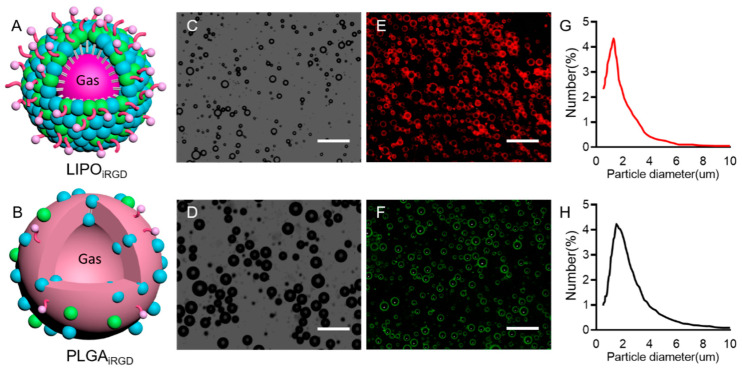
Schematic diagram of LIPO_iRGD_ and PLGA_iRGD_ and in vitro US imaging. Schematic diagrams of LIPO_iRGD_ (**A**) and PLGA_iRGD_ (**B**). Bright-filed photograph of LIPO_iRGD_ (**C**) and PLGA_iRGD_ (**D**). Fluorescent micrograph of DiI-labeled LIPO_iRGD_ (**E**) and DiO-labeled PLGA_iRGD_ (**F**). Average size and distribution of LIPO_iRGD_ (**G**) and PLGA_iRGD_ (**H**). Scale bar = 10 μm.

Schematic diagrams of the two types of targeted MBs were presented in Figure 1A,B. Microscopic image showed that the two targeted MBs had similar spherical shapes and good dispersion (Figure 1C,D). Figure 1E,F show the fluorescence images of DiI-labeled LIPO_iRGD_ and DiO-stained PLGA_iRGD_, indicating that iRGD are successfully coupled to the surface of MBs. Figure 1G,H show the typical size distributions of the two targeted and control MBs. The average sizes of the two targeted MBs were similar to those of the control MBs, which were 1.21 µm and 1.29 µm, respectively (*p* > 0.05).

### 3.2. In Vitro Imaging Performance of the Targeted MBs

**Figure 2:**
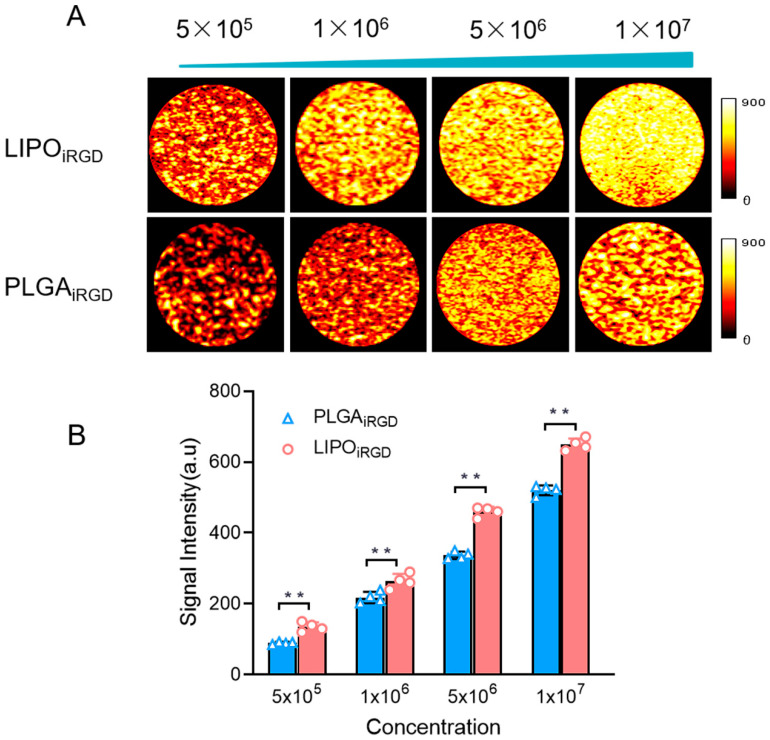
Characterization of LIPO_iRGD_ and PLGA_iRGD_. (**A**) Representative ultrasound contrast images of LIPO_iRGD_ and PLGA_iRGD_ at the concentrations from 5 × 10^5^ MBs/mL to 1 × 10^7^ MBs/mL. (**B**) Quantitative analysis of US signal intensities of LIPO_iRGD_ and PLGA_iRGD_. Data were analyzed by independent sample *t*-test (** *p* < 0.05 ).

To confirm the imaging performance of the two targeted MBs, we evaluated their imaging performance at four different concentrations in vitro. Figure 2A revealed that the imaging signal intensity of the two types of targeted MBs in the contrast mode increased along with the increase in the concentrations, achieving the strongest signal enhancement at 1 × 10^7^ MBs/mL. It is notable that lower signal intensities were observed for the PLGA_iRGD_ than LIPO_iRGD_ at the same concentrations (Figure 2B). The average signal intensities of the targeted PLGA_iRGD_ than LIPO_iRGD_ were 520.25 ± 13.89 a.u and 649.75 ± 16.78 a.u, respectively.

### 3.3. Binding Affinity of the Two Targeted UCAs to bEnd.3 Cells

**Figure 3:**
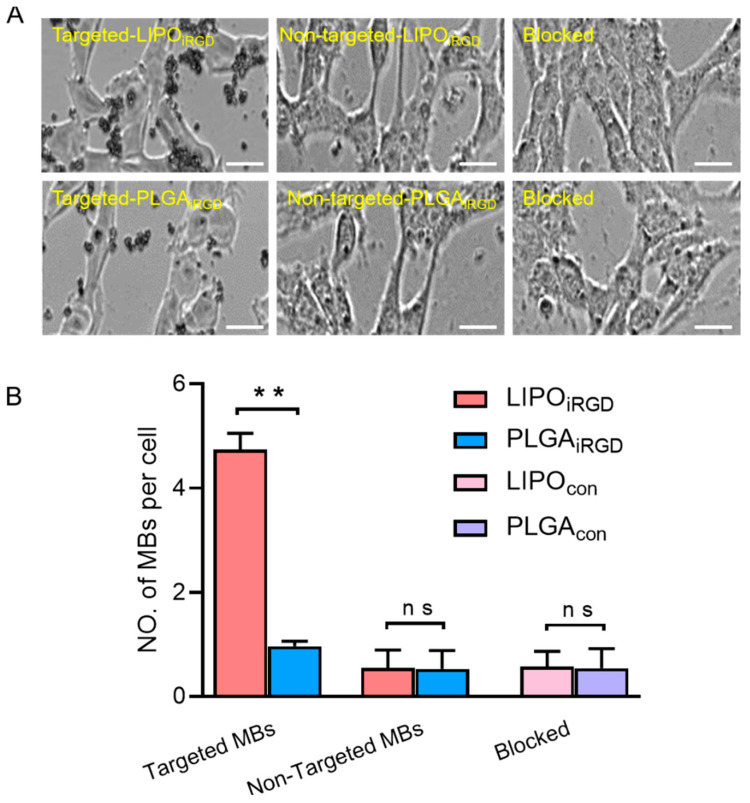
Binding ability of MBs to bEnd.3 cells. (**A**) In vitro cell binding of LIPO_iRGD_ and PLGA_iRGD_ to bEnd.3 cells. Blocked group indicates the bEnd.3 cells were pre-blocked with free anti-av antibody. (**B**) Quantitative analysis of the number of three groups of LIPO_iRGD_ and PLGA_iRGD_ that adhered onto bEnd.3 cells from five random view fields (** *p* < 0.01, *n* = 5). Scale bar = 10 μm. Data were analyzed by independent sample *t*-test (** *p* < 0.05, ns means *p* > 0.05).

Next, the binding affinity of the two targeted UCAs was examined by incubating them with bEnd.3 cells. Figure 3A showed white light microscopic images of the targeted UCAs binding onto the bEend.3 cells. From these pictures, we can see that a large number of targeted LIPO_iRGD_ bind to bEnd.3 cells (Figure 3A), achieving 4.73 ± 0.31 targeted LIPO_iRGD_ per cell, while significantly less targeted PLGA_iRGD_ could bind to bEnd.3 cells, with 0.96 ± 0.09 targeted PLGA_iRGD_ per cell. By contrast, hardly non-targeted LIPO_iRGD_ and PLGA_iRGD_ were observed to be able to bind onto the bEnd.3 cells. Notably, pre-blocking with free anti-av antibody significantly decreased both the number of targeted PLGA_iRGD_ and the LIPO_iRGD_ binding on bEnd.3 cells. Considering the significantly high cell-binding capability of LIPO_iRGD_ with bEnd.3 cells over PLGA_iRGD_, we used LIPO_iRGD_ in the ultrasound molecular imaging experiments.

### 3.4. Magnetic Resonance Imaging Detection

**Figure 4:**
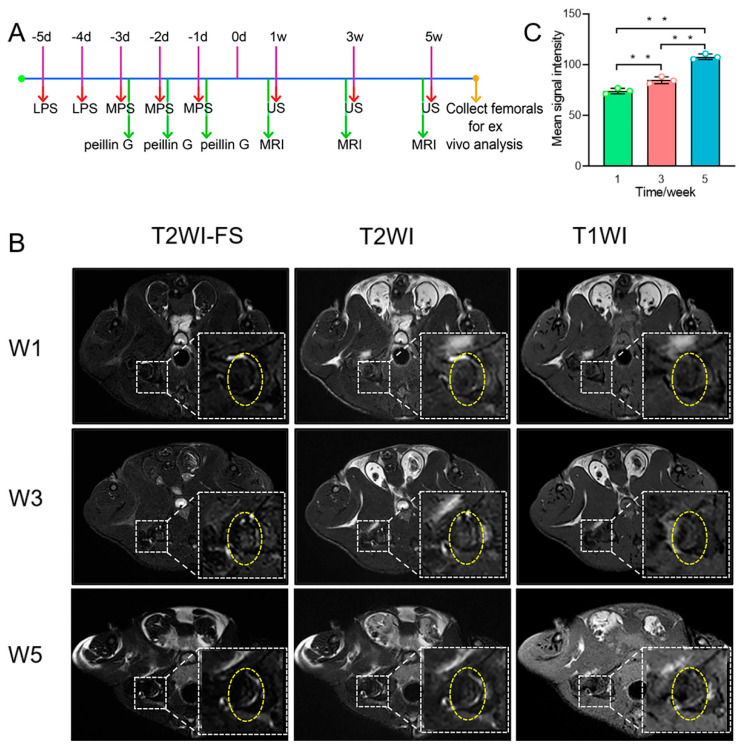
The establishment of ONFH model and MRI evaluation. (**A**) Flow diagram summarizes experimental design of the establishment of ONFH model. (**B**) The model of ONFH was monitored by MRI with three sequences (T2WI-FS, T2WI and T1WI) at 1 week, 3 weeks and 5 weeks. (**C**) Quantitative analysis of signal intensities for the T2WI. Data were analyzed by one-way ANOVA, and multiple comparisons of LSD were chosen as post hoc tests (** *p* < 0.05).

Figure 4A shows the schematic diagram of the timeline for the establishment of the ONFH model. To evaluate the successful establishment of the ONFH model, the bilateral femoral heads of rats were examined by MRI at 1 week, 3 weeks and 5 weeks. MR examination after 1 week showed that the hip joint space was normal, and the femoral head was smooth and intact without deformation (Figure 4B). Three weeks later, signs of osteonecrosis of the femoral head were observed by the T2WI fat compression sequence, showing a slightly higher signal intensity, and T1WI showed a slightly linear low signal. After 5 weeks, MR examination showed that osteonecrosis of the femoral head was aggravated, and the femoral head was not smooth. T2WI showed moderate and slightly higher signal intensities, and T1WI showed an uneven low signal intensity. Quantitative analysis of the ROI region from T2WI images showed that the mean signal intensities were 74.03 ± 2.82 for 1-week ONFH rats, 84.67 ± 3.46 for 3-week ONFH rats and 107.81 ± 2.83 for 5-week ONFH rats (Figure 4C). These results showed the ONFH model was successfully established, and the osteonecrosis of the femoral head can be evaluated by MRI.

### 3.5. In Vivo Ultrasound Molecular Imaging

**Figure 5:**
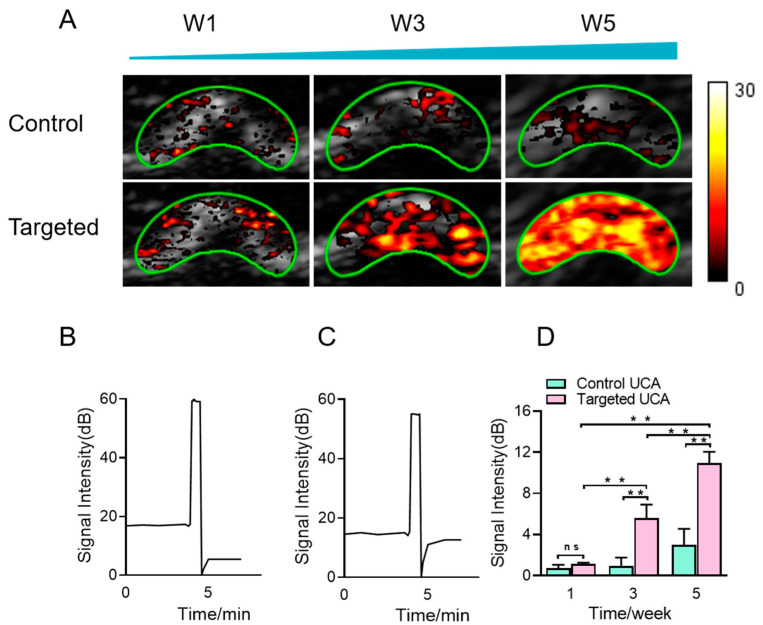
Ultrasound molecular imaging of ONFH in vivo. (**A**) Targeted and control MBs using for ultrasound molecular imaging in rat ONFH model (green borders: ROI borders) in rats. Color-targeted contrast signal was overlaid on gray brightness-mode (B mode) image. (**B**,**C**) Schematic diagram of targeted MBs and control MBs pre- and post-destruction. (**D**) Quantitative analysis of signal intensities for the targeted and control MBs. Data were analyzed by one-way ANOVA, and multiple comparisons of LSD were chosen as post hoc tests (** *p* < 0.05, ns means *p* > 0.05).

Non-invasive ultrasound molecular imaging was performed on ONFH model rats in vivo at the 1st, 3rd and 5th weeks, revealing significant enhanced ultrasound molecular imaging signals at the 3rd and 5th weeks (Figure 5A). Representative diagrams of the destruction/reperfusion process for iRGD-targeted MBs and control MBs at the 3rd week are provided in Figure 5B,C, confirming the signal difference. Quantitatively, at the 1st week, the signal intensity of the LIPO_iRGD_ experimental group was 1.13 ± 0.13 dB, while that of the non-targeted control group was 0.73 ± 0.31 dB. There was no statistically significant difference between the two groups (*p* > 0.05), indicating that there was no necrosis of the femoral head (Figure 5D). At the 3rd week, the signal intensity of the targeted group was 5.60 ± 1.31 dB, while that of the control group was 0.94 ± 0.81 dB. There was a significant difference between the two groups (*p* < 0.05) (Figure 5D). MRI also revealed that the rats’ femoral head was necrotic. At the 5th week, the signal intensity in the experimental group achieved 10.9 ± 1.15 dB, significantly higher than that of the control group with 2.98 ± 1.58 dB signal intensity (*p* < 0.05) (Figure 5D). More obvious necrotic and aggravated could be observed in the femoral head by MRI. Notably, with the elongation of time from one week to five weeks, the increasing trend in the signal intensity was significantly obvious in the experimental group compared to the control group (*p* < 0.05). 

### 3.6. Immunofluorescence Staining

**Figure 6:**
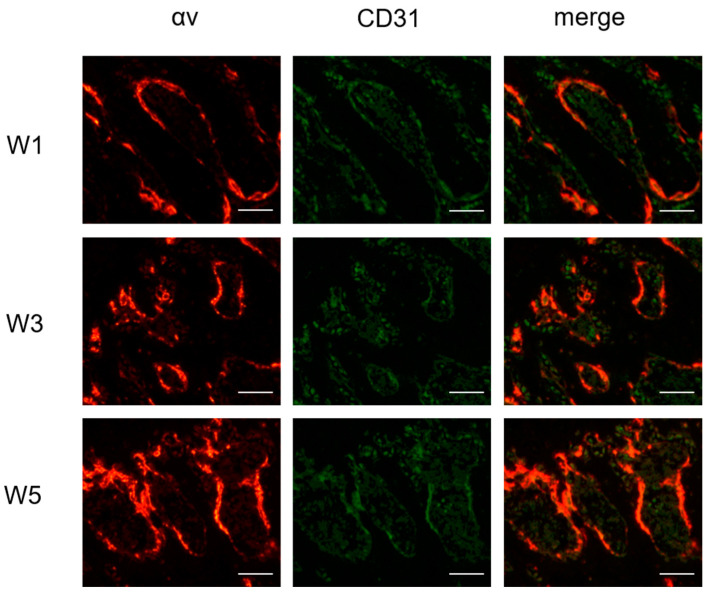
Immunofluorescence staining assay for av and CD31 expression on the ONFH section. Integrin α_v_ and blood vessel in ONFH were stained with fluorescence-labeled anti-α_v_ and CD31 antibodies. Immunofluorescence images of integrin α_v_ staining red (first column), immunofluorescence images of CD31 blood vessel staining green, and images obtained by merging (red and green) (third column). Bar = 100 μm.

To confirm the results of the ultrasound molecular imaging examination by LIPO_iRGD_, the femoral heads at the 1st, 3rd and 5th weeks were collected, and the expression of CD31 and integrin α_v_ were detected using the immunofluorescence method (Figure 6). From these images, we can see that the expression of α_v_ (red) and CD31 (green, a marker for vascular endothelial cells) endothelial markers can be co-located, confirming the existence of integrin α_v_β_3_ on the vascular endothelial cells of the ONFH. In addition, similar to CD31-positive cells, the integrin α_v_β_3_-positive cells at the 1st, 3rd and 5th weeks had rats gradually increase the severity of ONFH. Thus, the results of immunoblotting and histological evaluation of SIONFH at the 1st, 3rd and 5th weeks were related to the intensity of the ultrasonic signals obtained by α_v_β_3_ integrin-targeted LIPO_iRGD_.

## 4. Discussion

The early clinical symptoms of ONFH are not obvious and difficult to identify [6,36]. USMI shows great potential for early diagnosis of ONFH due to its unique advantages. It can non-invasively evaluate the molecular or cellular phenotype of the disease and provide spatial and temporal information on complex disease processes at the molecular level [37,38,39]. In the early stages of osteonecrosis of the femoral head, there are pathological changes in the blood vessels [8,26,40,41]. With the help of the strong backscattering ability of the UCA, the subchondral intraosseous vascular signal can be clearly observed. Integrin α_v_β_3_ is overexpressed in neovascularization but not expressed or minimally expressed in unaffected vessels [31,42,43], which provides a chance for USMI to detect SIONFH. 

In our study, we designed two kinds of targeted UCA, PLGA_iRGD_ and LIPO_iRGD_. PLGA_iRGD_ showed lower contrast signal intensities than LIPO_iRGD_ at the same bubble concentrations. It can be attributed to the more hard shell structure for PLGA_iRGD_, which was mainly made of PLGA polymer [44,45]. Additionally, we found there were significantly less targeted PLGA_iRGD_, which could bind onto the bEnd.3 cells. It is reasonable that there were less iRGD peptides coated on the PLGA_iRGD_ than LIPO_Irgd_ [33]. When taking the lower contrast signals and worse affinity with bEnd.3 cells, we only applied targeted LIPO_iRGD_ in our ultrasound molecular imaging for detecting SIONFH.

In our study, the differential signal intensity of targeted enhancement gradually increased with the severity of osteonecrosis of the femoral head, indicating that the expression of integrin α_v_β_3_ gradually increased. This change is consistent with the change in the trend of neovascularization during the repair of osteonecrosis of the femoral head [41,46,47]. In fact, our immunofluorescence results further confirmed the change in integrin α_v_β_3_ expression in ONFH, and the red α_v_β_3_ expression increased gradually. During the whole experiment, the expression of integrin α_v_β_3_ reached its highest level in the 5th week of ONFH but changed very little in the 1st week.

This study has several limitations. First, the ultrasonic diagnostic instrument used in this study is suitable for examining the human body but is not designed for small live animals. The relatively low acoustic transmitted frequency will decrease the imaging effect. Second, although integrin α_v_β_3_ is of great significance for the early diagnosis of SIONFH, targeted contrast agents anchoring two or more molecular biomarkers may be more advantageous than single targeted contrast agents [48,49]. The former can increase the accuracy of disease diagnosis by increasing the number of ultrasonic backscatter signals. In addition, the ultrasound imaging concentration of targeted LIPO_iRGD_ for SIONFH needs to be further optimized to improve the sensitivity of targeted LIPO_iRGD_ in SIONFH angiogenic molecular imaging.

## 5. Conclusions

In summary, our research shows that LIPO_iRGD_ targeting integrin α_v_β_3_ can be used to improve the contrast and resolution of ultrasound imaging in vivo, thus providing an effective and novel approach for early diagnosis of SIONFH. Our study, for the first time, proved that USMI can evaluate the occurrence and development of SIONFH at the molecular level.

## Data Availability

Not applicable.
